# Natural Food for Sarcopenia: A Narrative Review

**DOI:** 10.21315/mjms2022.29.4.4

**Published:** 2022-08-29

**Authors:** Nurul Syahidah Mohd Nazri, Divya Vanoh, Kah Leng Soo

**Affiliations:** Nutrition and Dietetics Programme, School of Health Sciences, Universiti Sains Malaysia, Kelantan, Malaysia

**Keywords:** food, vegetables, fruits, dietary pattern, sarcopenia

## Abstract

Sarcopenia is a syndrome characterised by progressive loss of skeletal muscle mass and strength. Proper nutrition is essential for management of sarcopenia. Thus, this article aims to review the association between dietary pattern or food groups consisting of natural food and sarcopenia. A literature search was performed using four databases namely PubMed, Scopus, Sage and ScienceDirect. The search terms used were ‘fruits’, ‘vegetables’, ‘egg’, ‘fish’, ‘chicken’, ‘protein food’, ‘*ulam*’, ‘fresh herbs’, ‘sarcopenia’, ‘elderly and ‘older adults’. A total of 18 studies were included in the final review. Adherence to Mediterranean and Japanese dietary pattern were associated with lower prevalence of sarcopenia whereas Western dietary pattern was significantly associated with higher risk of sarcopenia. For food groups, there is a significant association between dietary protein intake and sarcopenia. There are also significant associations between the intake of vegetables, fruits or both vegetables and fruits, and lower risk of sarcopenia. Consumption of natural food comprising of high-quality protein, fruits and vegetables have been associated with protection against muscle wasting and sarcopenia. Therefore, it is possible that a well-planned diet may works just as effectively as or possibly better than individual nutrient supplements for the prevention and treatment for sarcopenia among older adults.

## Introduction

The term ‘sarcopenia’ (Greek *sarx* or flesh and *penia* or paucity) was coined by Irwin Rosenberg which described the age-related muscle mass deterioration and eventually the term used to indicate the co-occurrence of skeletal muscle mass and strength loss among older adults (1). With the increased understanding on sarcopenia, the European Working Group on Sarcopenia in Older People (EWGSOP) had developed a practical clinical definition for sarcopenia which is ‘a syndrome characterised by progressive and generalised loss of skeletal muscle mass and strength with the risk of adverse outcomes such as physical disability, poor quality of life and death’ (2). The aging process had contributed to exceptional physical changes and one of the changes is gradual loss of skeletal muscle mass (3). Healthy adults may lose around 8% of muscle mass every 10 years after the age of 40 years old which may lead to a loss of an average of 24% of muscle between 40 years old and 70 years old. This further and speed up to 15% per decade after the age of 70 years old (1).

The prevalence of sarcopenia is greatly varied in different countries depending on the method of measurement, the population and the diagnostic criteria. A systematic review by Shafiee et al. (4) which includes studies that reported the prevalence of sarcopenia in healthy adults aged ≥ 60 years old using the EWGSOP, the International Working Group on Sarcopenia (IWGS) and Asian Working Group for Sarcopenia (AWGS) definition found that the prevalence of sarcopenia was 10% in men and 10% in women, respectively. The prevalence was higher among non-Asian than Asian individuals in both genders. According to EWGSOP, the criteria for sarcopenia are low muscle strength, low skeletal muscle mass or quality and low muscle performance (5). But sarcopenia diagnosis among Asian population might need some special considerations due to the differences in anthropometry, culture and lifestyle as compared to Western contemporaries as Asian population are having higher adiposity and less mechanised, relatively smaller body size and more physically active lifetimes compared to Western communities (6). Therefore, AWGS proposed a diagnostic algorithm in 2019 based on Asian data which resembled EWGSOP 2019 but with clearly defined cut-offs for individual diagnostic components. According to AWGS 2019, low muscle strength is defined as handgrip strength < 28 kg for men and < 18 kg for women; criteria for low physical performance are 6 m walk < 1.0 m/s, short physical performance battery score ≤ 9-time or 5-time chair stand test ≥ 12 sec (7). AWGS 2019 also introduces ‘possible sarcopenia’ which is defined by either low muscle strength or low physical performance. This condition will enable earlier lifestyle interventions.

There are several risks factors that may contribute to sarcopenia development including muscle activity reduction (e.g. immobilisation, sedentary lifestyle, prolonged bed rest and hospitalisation), diseases (e.g. chronic inflammatory diseases, malignancies, endocrine disorder and advanced organ failure) and nutrition (1). The role of nutrition is equally important especially on muscle health by influencing myocyte homeostasis and energy metabolism. Nutrient intake especially energy and/or protein intake might be inadequate due to malabsorption, use of anorexigenic drugs or gastrointestinal disorders (8). On top of that, the physiologic changes together with changes in dietary habits throughout adulthood had also consequently led to malnutrition. Data had shown that the energy intake had been decreasing approximately by 600 kcal in women and 1,330 kcal in men between the ages of 20 years old and 80 years old (2). It is also found that older adults are at a higher risk of inadequate protein intake as compared to their younger counterparts (9). Apart from proteins, there are many other nutrients that are essential for the maintenance of muscle mass including essential amino acids, creatine monohydrate, omega-3 polyunsaturated fatty acids (PUFAs), amino acid metabolites, folic acid, magnesium, antioxidants, vitamin D and vitamin B6 (2).

Some studies had been done to study the association between sarcopenia and natural foods. According to the European Union guidelines, natural food claims is only allowed when the food contains natural ingredients or when the product has no chemical additives [Regulation (EC) No. 1924/2006, Regulation (EC) No. 1047/2012] (10). The United States Department of Agriculture (USDA) specified that to claim a food as natural food, it must not have artificial flavouring and be minimally processed (10). Whole foods are also sometimes labelled as natural foods. A whole food such as fruits and vegetables has not been processed and contain only one ingredient which is themselves. One of the common natural foods that had been found to be associated with sarcopenia is vegetables and fruits. A recent study by Koyanagi et al. (11) found that women with the highest quantile of fruits consumption was associated with 40% lower odds for sarcopenia but no significant association was found among men.

The study also found that vegetables consumption was not significantly associated with sarcopenia. On the other hand, another study by Kim et al. (12) reported that men in the highest quantile of vegetables, fruits and both vegetables and fruits consumption demonstrated a lower risk of sarcopenia while only high consumption of fruits demonstrated lower risk of sarcopenia in women. Some studies also investigated the association between sarcopenia and dietary pattern. A lot of studies showed that a Mediterranean dietary pattern or a diet with a predominant intake of vegetables, fruits, protein from legumes and omega-3 fatty acids might have the potential to reduce the risk of sarcopenia among older adults. A cross-sectional study among 2,570 women aged 18 years old–79 years old by Kelaiditi et al. (13) have reported that a high adherence to the Mediterranean dietary pattern was significantly associated with increased muscle mass and legs explosive power (LEP). A review by Granic et al. (14) emphasise the beneficial effects of the Mediterranean dietary pattern towards the loss of muscle strength and muscle mass. However, the authors also suggest that there need to be a harmonise methods for defining dietary models such as Mediterranean diet (MedDiet) versus healthy eating index and also specially designed studies on different older adults’ populations with a longer follow-up study to reach a higher level of evidence. Therefore, this article reviews the association between dietary pattern or food groups consisting of natural food with sarcopenia in Asian and non-Asian countries.

## Methods

### Search Strategy

The search for published research papers related to natural food for sarcopenia were conducted using four databases which include PubMed, Scopus, Sage and ScienceDirect for articles published from 2010 to 2020 by using PRISMA method (15). The review includes only cross-sectional, prospective cohort and case control studies. Multiple search terms were combined using the Boolean function of ‘OR’ and ‘AND’ for searching the articles. The medical library subject heading terms used to search the related articles were ‘fruits’ OR ‘vegetables’ OR ‘egg’ OR ‘fish’ OR ‘chicken’ OR ‘protein food’ OR ‘*ulam*’ OR ‘fresh herbs’ AND ‘sarcopenia’ AND ‘elderly OR older adults’.

### Inclusion Criteria

Studies chosen for this review investigated the association between natural foods and sarcopenia among older adults. Natural foods are the food that have no added artificial colours or flavourings and have fresh ingredients which are minimally processed. Only cross-sectional, cohort studies and case-control studies were included in this review.

### Exclusion Criteria

Articles were excluded if investigating on supplementation of herbs, vitamin, minerals and protein such as protein powder, whey protein, soy-bean powder, protein bar, protein cereal or amino acid supplementation. Studies that do not include natural foods or natural ingredients or has chemical additives were excluded. Studies with subjects who were non-sarcopenic were excluded as well. Articles published in languages other than English were excluded as well.

### Study Selection

The prime literature search was conducted by the authors. The duplicate articles were removed. Hand search was conducted to confirm that the duplicate articles were removed. Papers included for this study was chosen based on the title and abstract followed by retrieval of full article from the database. Relevant articles were downloaded and assessed for eligibility.

### Data Organisation and Reporting

The final articles selected for the review were read thoroughly and each article was summarised according to the country where the study was conducted, study design, sample size, age, prevalence of sarcopenia, type of food or food groups or dietary pattern and findings of the study. The studies were reported according to the PRISMA guidelines. Table 1 explained the processes involved in the searching of the articles to be included in the review.

## Results

### Study Selection

A total of 6,210 records were identified through four electronic databases searching. About 36 duplicates were removed and the screening were done to the remaining articles based on title. A total of 29 articles qualified for the full text review. However, 11 articles had to be excluded from the review because three were not study on natural foods, one was a pilot study, six did not consider sarcopenia as study outcome and one was conducted among cancer patients. Thus, a total of 18 studies were included in the final review (11–12, 14, 16–30). Nine of the studies were conducted in Asian countries; three in China (16–18), five in Korea (12, 19–21, 29) and one in Japan (22). Eight studies were conducted in non-Asian countries whereby one was respectively conducted in Italy, United Kingdom, Netherlands and Poland (23), three in Iran (24–26), one in Finland (27) and three in the United Kingdom (14, 28, 30). One of the study were conducted in both Asian and non-Asian countries including China, Ghana, India, Mexico, Russia and South Africa (11). Nine studies (14, 17–18, 23–27, 30) had sample size of less than 1,000 people, five studies (12, 20–22, 28) had sample size between 1,000 and 3,000 people and another four (11, 16, 19, 29) had a sample size of > 3,000 people. As stated in methodology, the studies included in this review had cross-sectional, prospective cohort and case-control study design. The duration of the included studies ranged from a minimum 5 months to a maximum of 5 years.

### Association between Different Types of Foods or Dietary Patterns and Sarcopenia

A total of six studies measured the association between dietary patterns with sarcopenia (14, 16, 22, 24–26). Among the studies, one studies emphasised the Mediterranean dietary pattern (27), two studies focused on the Western dietary pattern (14, 24) and one study included both Mediterranean and Western dietary patterns (25). In addition, one study assessed Japanese dietary pattern (22) whereas one measured a combination of vegetables-fruits, snack-drinks-milk products and meat-fish dietary pattern (16). Another 12 studies measured the association between different types of foods or food groups with sarcopenia where three studies measured vegetable and fruits (11–12, 28), four studies assessed effect of protein food such as meat, milk, fish and eggs intake on sarcopenia (17–18, 27, 30), one study investigated the effect of coffee intake on sarcopenia (20), two studies measure dietary fibre intake (21, 23) whereas another two studies (19, 29) included multiple food groups. The study by Yoo et al. (19) investigated the association between protein, vegetable/fruit and dairy products intake whereas Lim (29) measured association between cereals, potato and starches, sugars and sweeteners, nuts and seeds, vegetables, mushroom, meats, milks, fruits, eggs, fish and shellfish, oil and fat, beverages with sarcopenia.

## Discussion

### Association between Dietary Pattern and Sarcopenia

A dietary pattern can be defined as the quantity, variety or combination of different foods and beverages in a diet and the frequency which are habitually consumed (31). Dietary pattern is important as it summarises the total diet which take into account the food that are consumed in complex combinations with interactions and synergies between dietary constituents and the balance between the components of protective and risk foods which may be important to find the association between diet and diseases (32). Nutritional epidemiology traditionally focused on the association of diseases with specific nutrients or food. However, the single-nutrient approach might fail to take into consideration the complicated interaction among nutrients and the potential confounding by the individual’s eating pattern as people eat a variety of foods with a complex combinations of nutrients (33). Therefore, an increasing number of research had been done using dietary pattern to characterise a population’s dietary intake in order to examine potential association between these patterns and health.

One of the most popular dietary patterns is MedDiet which is recognised as a healthy dietary pattern. All the studies included in this review found that greater adherence to MedDiet is associated with lower odds of sarcopenia (24–25). The MedDiet was first defined as being low in saturated fat and high in vegetable oils by Ancel Keys during the 1960s (34). However, the study of the MedDiet has advanced over the past several decades and the definition originally introduced by Keys has evolved and varied. MedDiet is characterised by a high consumption of plant-based foods, the use of olive oil as the main source of fat, a low intake of red meat and other processed foods and moderate consumption of wine during meals (35). Greater adherence to MedDiet has been found to be related to a reduced risk of causes of mortality, as well as lower incidence of mortality from cardiovascular diseases, type 2 diabetes, certain types of cancer and neurodegenerative diseases (36).

The beneficial effects of MedDiet on health might be related to several factors. One of the factors is higher fruits and vegetables content which are sources of antioxidants for reducing inflammation as well as reducing oxidative stress as the major mechanism associated in the pathogenesis of sarcopenia among older adults (26). Moreover, higher adherence to the MedDiet was associated with lower percentage of energy coming from total fat and saturated fatty acid (SFA), higher protein intake (as a percentage of energy) and increased ratio of monounsaturated fatty acid (MUFA) to SFA which indicate that subjects with high adherence to MedDiet had better nutrient profile and lower risk of nutrient deficiencies (36). In addition, MedDiet includes fish and nuts which are high in omega-3 fatty acids and vitamin D. Omega-3 fatty acids has anti-inflammatory actions which may significantly influence muscle function. On the other hand, vitamin D metabolites may influence muscle cell metabolism (26).

Another type of dietary pattern is Western dietary pattern. One of the studies included in this review found significant relationship between Western dietary pattern with the risk of prevalent sarcopenia (14) whereas two other studies found no association between Western dietary pattern with sarcopenia (24–25). The study by Granic et al. (14) reported that subjects in the Western diet group included higher proportion of butter, red meats, gravy, potatoes and sweets or desserts and this significantly increased the risk of sarcopenia. There are several processes that have been found to be the factors contributing to muscle wasting and loss of function due to Western dietary pattern including imbalance between muscle protein anabolism and catabolism, inflammation and production of pro-inflammatory cytokines (37) and inter- and intra-myocellular lipid accumulation (38) which might affect the quality of aged muscle of older adults. On the other hand, Mohseni et al. (24) found that there was no significant difference between muscle mass index and handgrip strength across tertiles of the Western dietary pattern. Similarly, Mohseni et al. (25) reported that there was no association between Western dietary pattern and sarcopenia. The lack of relationship between Western dietary pattern and sarcopenia is consistent with the Western dietary pattern derived in study by Hashemi et al. (26), which had high loadings for fast food, sweets, sugar and hydrogenated fat. The study suggested that the lack of association could be due to the presence of highly loaded food items such as soy products (as sources of iso-flavonoids) and tea which reduced the odds of sarcopenia by protecting muscle quantity and quality (26).

Another type of dietary pattern is Japanese dietary pattern. Many studies had found that traditional Japanese dietary pattern is associated with a lower risk of mortality and adverse health outcomes (39). The study included in this review by Suthutvoravut et al. (22) reported that low adherence to Japanese dietary pattern was associated with higher prevalence of sarcopenia. The traditional Japanese dietary cultures are collectively known as *Washoku*. According to Professor Kumakura Isao who is a professor at the Osaka National Museum of Ethnology, the guiding principles of *Washoku* refers to the presence of staple food (rice) which is complemented by a variety of side dishes, soup and pickles which is customarily eaten using chopsticks in wooden bowls known as *wan* (40). According to the Japanese Diet Index (JDI), there are seven adhering components (rice, miso soup, fish and shellfish, green and yellow vegetables, seaweed, pickles and green tea) and two non-adhering components (beef and pork, and coffee) are considered part of the traditional Japanese diet (41). *Washoku* benefits fully from the distinctive flavour (combination of taste, smell and tactile sensations) of each ingredients. The usage of chopstick lead to small bites together with the combination of foods inside the mouth seem to contribute to satiety. The relatively small portion size of the main and side dishes might help to avoid overeating (40).

The relationship between Japanese dietary pattern and sarcopenia might be related to increased consumption of high quality protein from both animal- and plant-products. One of the main components of Japanese dietary pattern is fish which is a good source of animal protein with low SFA, high in omega-3 fatty acids and vitamin D which are related to improved muscle mass and function (42) as well as older adults’s physical performance (43). Besides that, soybean (from miso soup) is a plant-based protein which is found to have the same protein digestibility-corrected amino acid (PDCAA) score as animal-based protein and might be related to increased muscle function (22). On top of that, the combination of animal and plant based protein might further enhance the postprandial muscle protein synthesis response (44).

### Association between Different Food Groups and Sarcopenia

It is known that energy intakes tend to decrease together with aging and poor protein intakes have been constantly reported among older adults with sarcopenia. All of the studies included in this review found that there are significant association between dietary protein intake with sarcopenia. Jyväkorpi et al. (27) found that sarcopenia is inversely associated with protein intake, Verlaan et al. (30) found lower protein intake among sarcopenic group as compared to non-sarcopenic group whereas both Xia et al. (17) and Yang et al. (18) found negative association between sarcopenia and consumption of meat, eggs and total protein intake. Yang et al. (18) also suggested that the effect of animal protein is greater than plant protein on increasing muscle mass which might be due to the fact that animal proteins can provide more energy and improve muscle function more efficiently. Another study by Yoo et al. (19) found that the proportion of individuals with a dietary protein intake below 0.91 g/kg/day was higher among the sarcopenic obesity group than in the control group. Protein is known to be one of the most important nutrients for older adults with low intakes been associated with higher losses of lean mass (19). Many researchers suggested that daily protein intake above the recommended dietary allowance (RDA) of 0.8 g/kg/d might be associated with higher physical performance, maintenance and increased muscle mass and decreased risk of physical disability (45). Therefore, a higher recommended protein intake of 1.0 g/kg/d–1.2 g/kg/d was recently proposed for healthy maintenance of muscle and up to 1.2 g/kg/d–1.5 g/kg/d for older adults with acute or chronic diseases (29). Besides protein, adequate energy intake is another factor which is related to healthy muscle mass. Limited energy intake affects protein synthesis by reducing the size of myofibrils, interfering with the protein kinase B (AKT)-dependent signalling pathway and activating the p38 signalling pathway (46–47). This is in agreement with the study by Okamura et al. (48) which demonstrated that adequate dietary intake was associated with lower risk of sarcopenia among older adults with type 2 diabetes.

Apart from protein, some studies also found the association between sarcopenia with vegetable and fruits intake. Kim et al. (12) found that the intake of vegetables, fruits and both vegetables and fruits was associated with lower risk of sarcopenia whereas Koyanagi et al. (11) found that fruits intake was associated with lower odds for sarcopenia but vegetables was not significantly associated with sarcopenia. Another study by Yoo et al. (19) found that the vegetables and fruits intake were significantly lower in the sarcopenic obesity group than in the control group. The study by Oh and Park (21) found that the sarcopenic group had lower intake of fruits and vegetables and those in highest tertile of fibre intake had lower odds of sarcopenia whereas Montiel-Rojas et al. (23) found that women above the median fibre intake had significantly higher skeletal muscle mass index (SMI) as compared to those below median fibre intake. Another study by Welch et al. (28) found significant associations between higher intakes of dietary antioxidants vitamin C, total carotene and specific carotenoids with improved sarcopenic indices of skeletal muscle. The study also found that vegetables were the most significant source of the antioxidants. The largest vegetable contributors to vitamin C were peppers, Brussels sprouts and broccoli carrots and spinach for carotene whereas vitamin E was supplied by avocado and mushrooms. Overall fruit was the greatest contributor to vitamin C intakes and whole grain cereals were the greatest contributor to vitamin E intake (28). Vitamin C is involved in the synthesis of collagen, carnitine and also retinol in protein metabolism, collagen formation and lipid oxidation which make vitamin C as a promising candidates for the prevention and treatment of age-related loss of muscle mass and function (49). Moreover, vitamin C and vitamin E had been found to have a roles as exogenous antioxidant and anti-inflammatory agents which might influence skeletal muscle and function.

Skeletal muscle’s aging process contributed to increased circulating cytokines and production of reactive oxygen species (ROS), harmful effects on synthesis of protein together with direct cellular damage of skeletal muscle fibres and DNA (50). Moreover, the proportion, quality and viscoelastic properties of the different types of collagen that form the important structural component of skeletal muscle cells, connective tissues and tendons might also be affected during aging process (51). With reduced endogenous antioxidant efficacy with aging and generation of ROS by skeletal muscle in the body, exogenous antioxidants might have important role for skeletal muscle health. Dietary antioxidants might help in the prevention and treatment of age-related loss of muscle mass and function through their roles as exogenous antioxidants and anti-inflammatory agents. Moreover, dietary carotenoids such as α-carotene, β-carotene, β-cryptoxanthin, lutein, lycopene and zeaxanthin might give protection against oxidative stress through their ability to quench singlet oxygen, scavenge free radicals, inhibit lipid peroxidation and modulate redox-sensitive transcription factors involved in the upregulation of pro-inflammatory cytokines (12).

Another study included in this review found association between coffee intake with sarcopenia. The study by Chung et al. (20) found that sarcopenia was significantly lower in people whose daily coffee consumption was at least three cups as compared to those with less than a cup. Meanwhile, the prevalence of sarcopenia was not significantly lower for persons who consumed one or two cups of coffee a day (20). This can be explained by the fact that coffee has phenolic compounds such as caffeic acid and chlorogenic acid that have strong antioxidant and anti-inflammatory activities as well as able to induce autophagy (52). As mentioned before, aging process cause mitochondrial dysfunction due to oxidative damage to mitochondrial DNA. Therefore, autophagy is important for proper renewal of mitochondria and maintenance of muscle mass (52). According to Pietrocola et al. (52), chronic coffee intake diluted in the drinking water of female mice stimulated autophagy in the liver, heart and skeletal muscle in a dose-dependent manner. Moreover, polyphenols which is the main antioxidant component of coffee have been found to be able to induce autophagy (53). Therefore, it is conceivable that coffee may lower the risk of sarcopenia due to its antioxidant properties by reducing oxidative stress of mitochondria of muscle cells.

The strength of this review is that it determines the importance of consuming good quality natural food for improvement in sarcopenia. In this review, the causes of sarcopenia related to food consumption have been highlighted which enable general practitioners to screen the dietary intake of older adults. The findings from this study have found that factors which lowers the risk of sarcopenia are adequate consumption of fruits, vegetables, dairy products, complex carbohydrate and protein (legumes, nuts, tofu, lean meat, fish and eggs). On the other hand, causes which increases risk of sarcopenia are excessive consumption of refine carbohydrate, saturated fat and cholesterol especially consumption of red meat, butter, desserts and refine carbohydrate. Thus, this study which highlights the understanding of the causes of sarcopenia will assist in improving overall health and quality of life of older adults. However, this study is not without limitation. Authors were unable to obtain more articles since Embase is not accessible in the author’s organisation. Thus, we needed to use the available databases which can provide more articles.

## Conclusion

In summary, the review suggests that healthy dietary pattern including Mediterranean and Japanese dietary pattern might give protection against sarcopenia due to the higher intake of vegetables, fruits, good quality protein and lower intake of fat. In addition, small portion of size of meal might help avoid overeating. On the other hand, Western dietary pattern has been found to increase the risk of sarcopenia. This review also suggests that a higher daily protein intake of 1.0 g/kg/d–1.2 g/kg/d is important for healthy maintenance of muscle and up to 1.2 g/kg/d–1.5 g/kg/d for older adults with acute or chronic diseases. Higher consumption of vegetables and fruits is also important for prevention and treatment against sarcopenia. Therefore, it can be concluded that it is possible that a well-planned diet may work just as effectively as or possibly better than individual nutrient supplements for the prevention and treatment for sarcopenia among older adults. Supplements such as protein with certain key micronutrients might be useful to those who are unable to follow a healthy diet due to several factors including cognitive decline, inability to prepare a meal, chewing or swallowing disabilities and are severely undernourished.

Future research must focus on exploring the effect of natural food on metabolite pathways affecting skeletal muscle.

## Figures and Tables

**Table 1:** Association between different types of foods or dietary patterns and sarcopenia

Author/Year	Country	Method	Sample size (*n*)	Age (years old)	Prevalence of sarcopenia (%)	Type of food/Food groups/Dietary pattern	Findings
Koyanagi et al. (2020) (11)	China, Ghana, India, Mexico, Russia and South Africa	Cross-sectional study	14,585	≥ 65	15.7	Vegetables and fruits	Compared to the lowest quantile, the highest quintile of fruit consumption was associated with a 40% lower odds for sarcopenia. Vegetable consumption was not significantly associated with sarcopenia
Chan et al. (2016) (16)	China	Prospective cohort study	3,957	≥ 65	7.3	Vegetables-fruits, snack-drinks-milk products (coffee, fast foods, nuts, french fries, milk and milk products, sweets and dessert, beverages) and meat-fish dietary pattern	Men with higher ‘vegetables-fruits’ and ‘snacks-drinks-milk products’ dietary pattern score had lower likelihood of being sarcopenic
Xia et al. (2016) (17)	China	Cross-sectional study	830	≥ 60	20.1	Meat, egg and total protein	Meat consumption (*P* = 0.0119), egg consumption (*P* = 0.0302) and the total protein consumption (*P* = 0.0302) were negatively related with sarcopenia
Yang et al. (2019) (18)	China	Case-control study	316	≥ 60	28.8	Meat, eggs and milk	There are significant differences in the prevalence of sarcopenia between the sarcopenic group and control with different dietary intake of meat, fish, eggs, and milk. The prevalence of sarcopenia is negatively correlated with the consumption of meat, eggs, and milk.
Yoo et al. (2020) (19)	Korea	Cross-sectional study	3,937	≥ 40	52.5 sarcopenic obesity	Protein, vegetable/fruit and dairy products intake	The proportion of individuals with a dietary protein intake below 0.91 g/kg/day was higher in the sarcopenic obesity group than in the control group in both age subgroups (middle-aged; *P* < 0.000, older aged; *P* = 0.0066). The vegetable and fruit intake were significantly lower in the sarcopenic obesity group than in the control group only in the middleaged subgroup (*P* < 0.001), whereas the dairy intake was not significantly different between the two groups
Chung et al. (2017) (20)	Korea	Cross-sectional study	1,781	≥ 60	7.01	Coffee (no distinction was made between caffeinated and decaffeinated coffee or between the individual types of coffee (boiled, filtered or instant)	Compared to those whose daily coffee consumption was < one cup per day, sarcopenia was significantly lower in people whose daily consumption was at least three cups, while the prevalence of sarcopenia was not significantly lower for persons who consumed one cup or two cups of coffee a day
Kim et al. (2015) (12)	Korea	Cross-sectional study	1,912	≥ 65		Vegetables and fruits	Dietary intake of vegetables (*P* = 0.026), fruits (*P* = 0.012) and both vegetables and fruits (*P* = 0.003) were associated with a significantly reduced risk of sarcopenia. In women, high consumption of fruits demonstrated a lower risk of sarcopenia
Lim (2020) (29)	Korea	Cross-sectional study	3,350	≥ 65	25.7	Cereals, potato and starches, sugars and sweeteners, nuts and seeds, vegetables, mushroom, meats, milks, fruits, eggs, fish and shellfish, oil and fat, beverages	The male sarcopenia group had significantly lower intakes of nuts and seeds, meats and milks whereas female sarcopenia group had significantly lower intake of fruits, milks and beverages compared to non-sarcopenia group
Oh and Park (2019) (21)	Korea	Cross-sectional study	1,527	≥ 50		Dietary fibre intake (fruits and vegetables)	When compared with the control, the sarcopenic group had significantly lower intake of fruits and vegetables. Those in the highest tertile of fibre intake had lower odds of sarcopenia (OR: 0.47 as compared with the participants in the lowest tertile
Suthutvoravut et al. (2020) (22)	Japan	Cross-sectional study	1,241	≥ 65	5.1	Japanese dietary pattern (comprising soybeans and soybean products, fish, vegetables, pickles, mushroom, seaweeds and fruits)	Men with the lowest tertile of dietary pattern 1 score (high loadings for fish, tofu, vegetables and fruits) had a higher likelihood of being sarcopenic. Low adherence to Japanese dietary pattern was associated with prevalence of sarcopenia in both genders
Montiel-Rojas et al. (2020) (23)	Italy, UK, Netherlands, Poland	Cross-sectional study	981	≥ 65		Dietary fibre intake	Women above the median fibre intake had significantly higher skeletal muscle mass index (SMI) compared to those below median fibre intake (*P* = 0.011). However, there was no significant impact of fibre intake on physical function outcomes (short-physical performance battery test and handgrip strength)
Mohseni et al. (2016) (24)	Iran	Cross-sectional study	250	45–75		Prudent dietary pattern (high in vegetables, vegetable oil, fish, dairy, legumes, nuts, animal protein and fruits) and Western dietary pattern (high in commercial beverage, hydrogenated fat, sugar, sweet snacks, potato, tea/coffee and refined grains).	Mean handgrip strength (*P* = 0.03) and gait speed (*P* < 0.01) were higher in the third tertile of the prudent dietary pattern compared with the first tertile. There was no significant difference between the third and first tertiles of the prudent dietary pattern in the mean muscle mass index. Gait speed was significantly lower among the participants in the third tertile of the Western dietary pattern compared with the participants in the first tertile. No significant differences were seen in muscle mass index and handgrip strength across tertiles of the Western dietary pattern.
Mohseni et al. (2017) (25)	Iran	Cross-sectional study	250	≥ 45	22	Western dietary pattern (high in commercial beverage, sugar and dessert, snacks, solid fat, potato, high fat dairy, legumes, organ meat, fast food, and sweets) and Mediterranean dietary pattern (high in olive, low-fat dairy, vegetable, fish, nut and vegetable oil)	The Mediterranean dietary pattern was inversely associated with sarcopenia, whereas no association was found with the Western dietary pattern
Hashemi et al. (2015) (26)	Iran	Cross-sectional study	300	≥ 55	18	Mediterranean dietary pattern (high olive oil, fruits, vegetables, fish and nuts intake)	Subjects in the highest tertile of the Mediterranean dietary pattern had a lower odds ratio for sarcopenia than those in the lowest tertile
Jyväkorpi et al. (2020) (27)	Finland	Cross-sectional study	126	≥ 80	21	Protein food (plant and animal protein from meat, milk, fish and eggs)	Sarcopenia status is inversely associated with total protein (*P* = 0.019), plant protein (*P* = 0.008) and fish protein (*P* = 0.041) intakes
Verlaan et al. (2017) (30)	UK	Cross-sectional study	132	≥ 65	50	Protein intake	The sarcopenic group consumed less protein/kg (−6%) as compared to the non-sarcopenic controls
Welch et al. (2020) (28)	UK	Cross-sectional study	2,570	18–79		Dietary antioxidant vitamins C, E and carotenoids intakes from fresh food	Higher vitamin C intake was associated with significantly higher indices of fat-free mass (FFM) and LEP, (*P* < 0.01–0.02). Intakes of total and individual carotenoids were significantly associated with indices of FFM and LEP whereas vitamin E was significantly associated with FFM only
Granic et al. (2019) (14)	UK	Prospective cohort study	751	≥ 85		Traditional Britsh dietary pattern (butter, red meat, gravy and potato)	A dietary pattern high in foods characteristic of a traditional British diet was associated with an increased risk of prevalent sarcopenia at baseline (*P* = 0.05) and 3-year follow-up (*P* = 0.003) even when overall protein intake was good
